# Susceptibility of primary ovine dorsal soft palate and palatine tonsil cells to FMDV infection

**DOI:** 10.3389/fvets.2024.1299379

**Published:** 2024-08-01

**Authors:** Morgan Sarry, Eve Laloy, Anthony Relmy, Aurore Romey, Cindy Bernelin-Cottet, Anne-Laure Salomez, Hélène Huet, Sara Hägglund, Jean-François Valarcher, Labib Bakkali Kassimi, Sandra Blaise-Boisseau

**Affiliations:** ^1^UMR VIROLOGIE, INRAE, École Nationale Vétérinaire d'Alfort, ANSES Laboratoire de Santé Animale, Université Paris-Est, Maisons-Alfort, France; ^2^AgroParistech, Paris, France; ^3^Host Pathogen Interaction Group, Section of Ruminant Medicine, Department of Clinical Science, Swedish University of Agricultural Sciences (SLU), Uppsala, Sweden

**Keywords:** foot-and-mouth disease virus (FMDV), persistence, cellular model, primary cells, sheep, palatine tonsils, dorsal soft palate

## Abstract

Foot and mouth disease (FMD) is a highly contagious viral disease affecting cloven-hoofed animals. This disease is one of the most important in animal health due to its significant socio-economic impact, especially in case of an outbreak. One important challenge associated with this disease is the ability of the FMD virus (FMDV) to persist in its hosts through still unresolved underlying mechanisms. The absence of relevant *in vitro* models is one factor preventing advancement in our understanding of FMDV persistence. While a primary bovine cell model has been established using cells from FMDV primary and persistence site in cattle, it appeared interesting to develop a similar model based on ovine anatomical sites of interest to compare host-pathogen interactions. Thus, epithelial cells derived from the palatine tonsils and the dorsal soft palate were isolated and cultured. Their epithelial nature was confirmed using immunofluorescence. Following monolayer infection with FMDV O/FRA/1/2001 Clone 2.2, the FMDV-sensitivity of these cells was evaluated. Dorsal soft palate (DSP) cells were also expanded in multilayers at the air-liquid interface to mimic a stratified epithelium sensitive to FMDV infection. Our investigation revealed the presence of infectious virus, as well as viral antigens and viral RNA, up to 35 days after infection of the cell multilayers. Further experiment with DSP cells from different individuals needs to be reproduced to confirm the robustness of the new model of persistence in multilayer DSP. The establishment of such primary cells creates new opportunities for FMDV research and analysis in sheep cells.

## 1 Introduction

Wild and domestic artiodactyls are susceptible to the highly contagious foot-and-mouth disease (FMD). Regarding its potential socio-economic effects in case of an outbreak, it is among the most significant animal diseases (1). Foot-and-mouth disease virus (FMDV), the FMD etiological agent, is a single-stranded positive RNA virus that belongs to the Aphtovirus genus within Picornaviridae family (2).

FMDV infection is characterized by the appearance of aphthae on epithelia, a state of lethargy, and loss of appetite (3). Domestic cattle, which are thought to be revelatory, exhibit the clinical signs quite readily, whereas the infection is less severe in small ruminants such as goat and sheep which act as disseminators (4). Although mortality in adult animals is typically low, it can be higher in young animals, particularly in piglets, lambs, and calves, due to acute myocarditis (5).

Up to 50% of ruminants remain infected after clinical recovery. Regardless of their particular FMD immune status, they become asymptomatic carriers (4, 6). Such healthy carriers constitute an obstacle to FMDV control because they represent a potential source of new recombinants and a threat to transmit FMDV to susceptible animals (5, 7–9). FMDV persistence is currently defined as the detection of infectious virus after 28 days post-infection. Mechanisms underlying FMDV persistence are still not fully understood more than 50 years after it was first described (10). The differential persistence of FMDV constitutes one of the gaps of knowledge that remains unsolved as there are currently no data to explain why FMDV persistence has been reported in ruminants but not in pigs (11, 12). The FMDV primary and persistent sites of infection have been identified over the past few decades and specifically, the dorsal soft palate and oropharyngeal tonsils in ruminants (4).

The lack of suitable study models is one barrier to furthering the understanding of FMDV persistence. Animal studies are expensive, involve ethical concerns, and call for huge infrastructure. Since the development of *in vitro* methods enabling the growth of the virus, various epithelial cell lines have served as study models (13–17). Among the standard cell lines used, Fetal goat tongue (ZZ-R127) cells, are the only one established from FMDV-sensitive tissue. In order to improve *in vitro* FMDV study, a multilayer cellular model derived from primary bovine dorsal soft palate (DSP), cultured at the air-liquid interface (ALI) has been developed by Hägglund et al. (18). It has been demonstrated that this *in vitro* DSP-ALI model allowed the establishment of a persistent FMDV infection and contributed to a better understanding of the transcriptional responses to FMDV infection (19). A similar model developed using porcine DSP cells has also been established (20).

Given the opportunities provided by such a model and the lack of a suitable ovine cell model, we isolated primary cells from ovine palatine tonsils (PT) and dorsal soft palate to create a multilayered culture model at the air-liquid interface. To study the acute and long-term FMDV infection in relevant ovine cells, we first characterized these cells and then infected them as monolayers then multilayers.

## 2 Materials and methods

### 2.1 Virus

The FMDV O/FRA/1/2001 Clone 2.2 (GenBank: OV121130.1) used in this study is a twice-plaque-purified viral clone derived from the O/FRA/1/2001 strain that was further propagated on BHK-21 cells (14).

### 2.2 Isolation of epithelial cells from the ovine palatine tonsils and dorsal soft palate

Epithelial tissue from the PT and DSP was collected immediately after slaughter of four sheep. Cells derived from the four sheep has been treated independently. Dissociation of the surface epithelium was performed to remove as much connective and muscle tissue as possible. Epithelial tissue was dissected and digested at 4°C overnight in incubation medium as detailed in Hägglund et al. (18).

### 2.3 Air-liquid interface multilayers

Cell culture at the air-liquid interphase was performed as described by Hägglund et al. (18). Ovine dorsal soft palate cells and palatine tonsils cells were thawed and immediately seeded on the rehydrated inserts at a density of 1 x 10^6^ cells per insert. After 4 weeks, when the cells formed a complete monolayer, the culture medium was removed from the upper compartment. The culture medium contained in the lower compartment was changed every 2 or 3 days during 5 weeks.

### 2.4 Cell characterization

The cellular expression of epithelial and mesenchymal markers such as cytokeratin and vimentin, as well as that of integrin α_V_β_6_, the specific receptor for FMDV, was analyzed after five cell passages in culture flasks, as described by Sarry et al. (20).

Cell multilayers were characterized as described by Hägglund et al. (18).

### 2.5 FMDV inoculation of cell monolayers

Ovine DSP and PT cells were propagated in culture flasks for four passages before being seeded in 48-well plates at a density of 1 × 10^5^ cells per well in DMEM complete medium. Once the cells formed a complete monolayer, two wells were trypsinized to count the cells and adjust the multiplicity of infection (MOI). Viral inoculum of FMDV O/FRA/1/2001 Clone 2.2 was diluted at MOI 10^−2^, MOI 10^−4^ and MOI 10^−6^ in a serum-free DMEM complete medium. Viral inoculum has not been removed from the inserts until the first sample collection. Infection follow-up was performed in the same way as described in Sarry et al. (20).

### 2.6 FMDV inoculation of multilayered cells

Ovine DSP cells were cultured on inserts for 5 weeks and were thereafter inoculated with FMDV O/FRA/1/2001 Clone 2.2 at MOI 10^−4^ or incubated with conditioned-medium. Infection follow-up was performed in the same way as described in Sarry et al. (20).

### 2.7 Culture supernatant analysis

Culture supernatants collected during infection were analyzed for infectious virus, FMDV 3D^pol^ antigen and 3D^pol^ RNA detection as described in Hägglund et al. (18).

## 3 Results

### 3.1 Ovine DSP and PT cells are *in vitro* cultivatable epithelial cells

**Figure 1:**
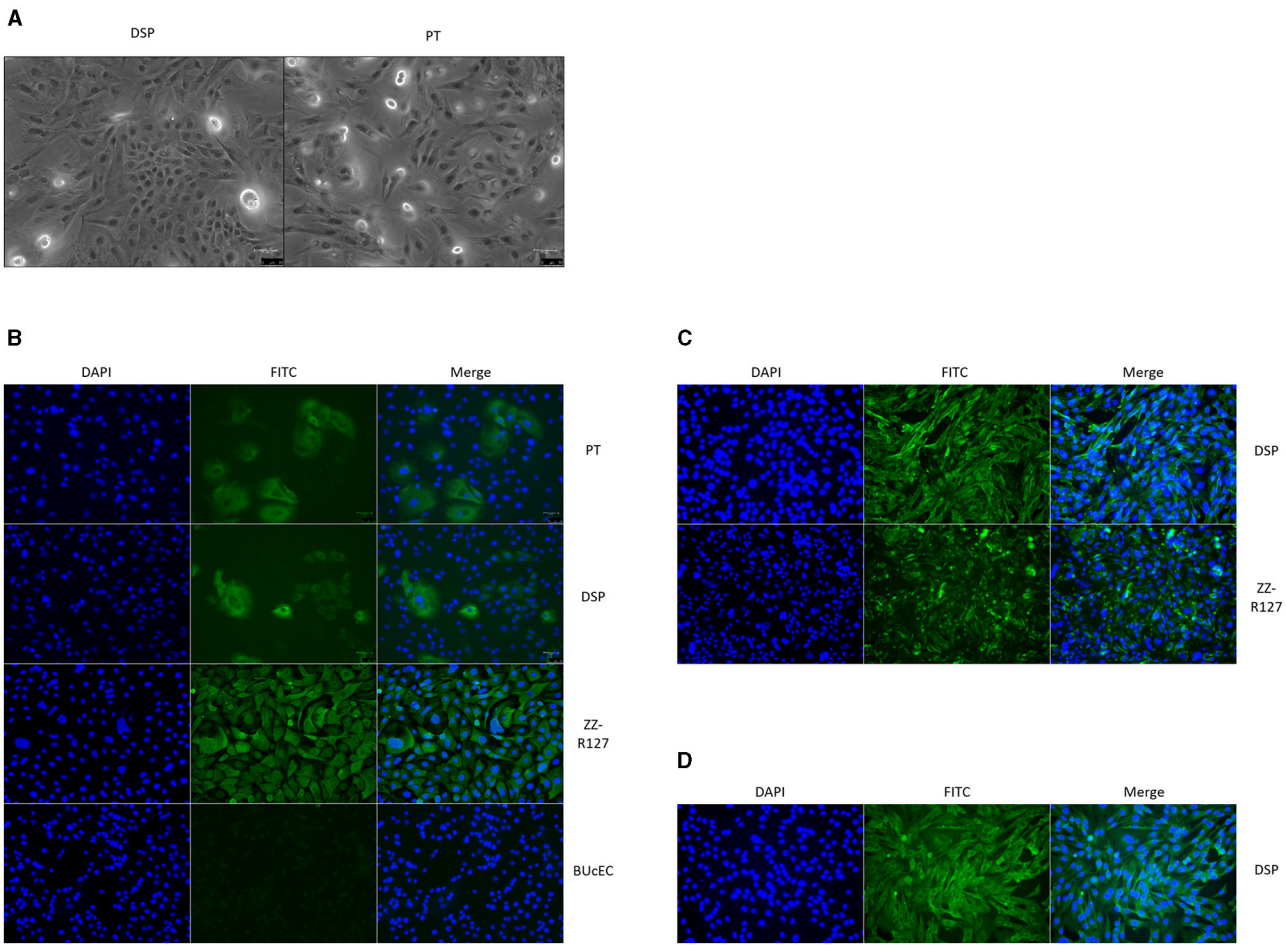
Ovine DSP and PT cells characterization. **(A)** DSP and PT morphological observation. DSP and PT cells were cultured in monolayers and were observed under brightfield microscopy (×100). **(B)** Cytokeratin staining. DSP and PT were cultured in monolayers on High ibiTreat Ibidi chamber slides. Cell nuclei were stained with Hoechst (blue) and cells were visualized by fluorescence microscopy using a DMi8 microscope. Staining with specific mouse cytokeratin antibody and a goat anti-mouse Alexa 488 (green). DSP staining were compared to ZZ-R127 epithelial cell line known to strongly express cytokeratin and BUcEC endothelial cell line known to not express cytokeratin. **(C)** Vimentin staining. DSP were stained with specific mouse vimentin antibody and a goat anti-mouse Alexa 488 (green). DSP staining were compared to ZZ-R127 epithelial cell line. **(D)** α_V_β_6_ integrin staining. DSP were stained with specific mouse cytokeratin antibody and a goat anti-mouse Alexa 488 (green).

Primary cells derived from ovine DSP and PT were isolated on culture plates, then propagated. The DSP cells were quite easier to detach during the trypsinization step than the PT cells and seem to develop slightly faster. Morphology of the PT and DSP cells was examined under a brightfield microscope (Figure 1A). We observed cells that resembled epithelial cells in shape, with polygonal or cobblestone morphologies and rather irregular contours (21).

After five passages cells were characterized by IF. Anti-cytokeratin immunostaining resulted in a positive signal that was lower than the one associated with the positive control based on ZZ-R127 cells. While almost all the ZZ-R127 cells were shown to express cytokeratin, only 30% of DSP cells and 40% of PT cells seem to be expressing this epithelial protein marker. Staining performed using bovine endothelial cells derived from umbilical cord (BUcEC), considered as a negative control for cytokeratin detection, was not associated with any signal as shown in Figure 1B. The secondary antibody alone did not generate fluorescence at similar conditions of fluorescent microscopy (data not shown).

Anti-vimentin immunostaining of ovine DSP indicates that a higher percentage of cells were expressing vimentin than cytokeratin (Figure 1C). Additionally, a more diffuse distribution of vimentin was found in the epithelial cell line ZZ-R127. The secondary antibody alone did not generate fluorescence at similar conditions of fluorescent microscopy (data not shown).

In order to make sure that DSP cells express integrin α_V_β_6_, the particular FMDV receptor needed to start an infection, a protein-specific antibody was used to mark DSP cells, before infection. With the help of this labeling, we were able to detect a signal that indicated that the integrin α_V_β_6_ was expressed significantly in our cells, as seen in Figure 1D. The secondary antibody alone did not generate fluorescence at similar conditions of fluorescent microscopy (data not shown).

### 3.2 Ovine DSP and PT monolayers are susceptible to type-O FMDV infection

**Figure 2:**
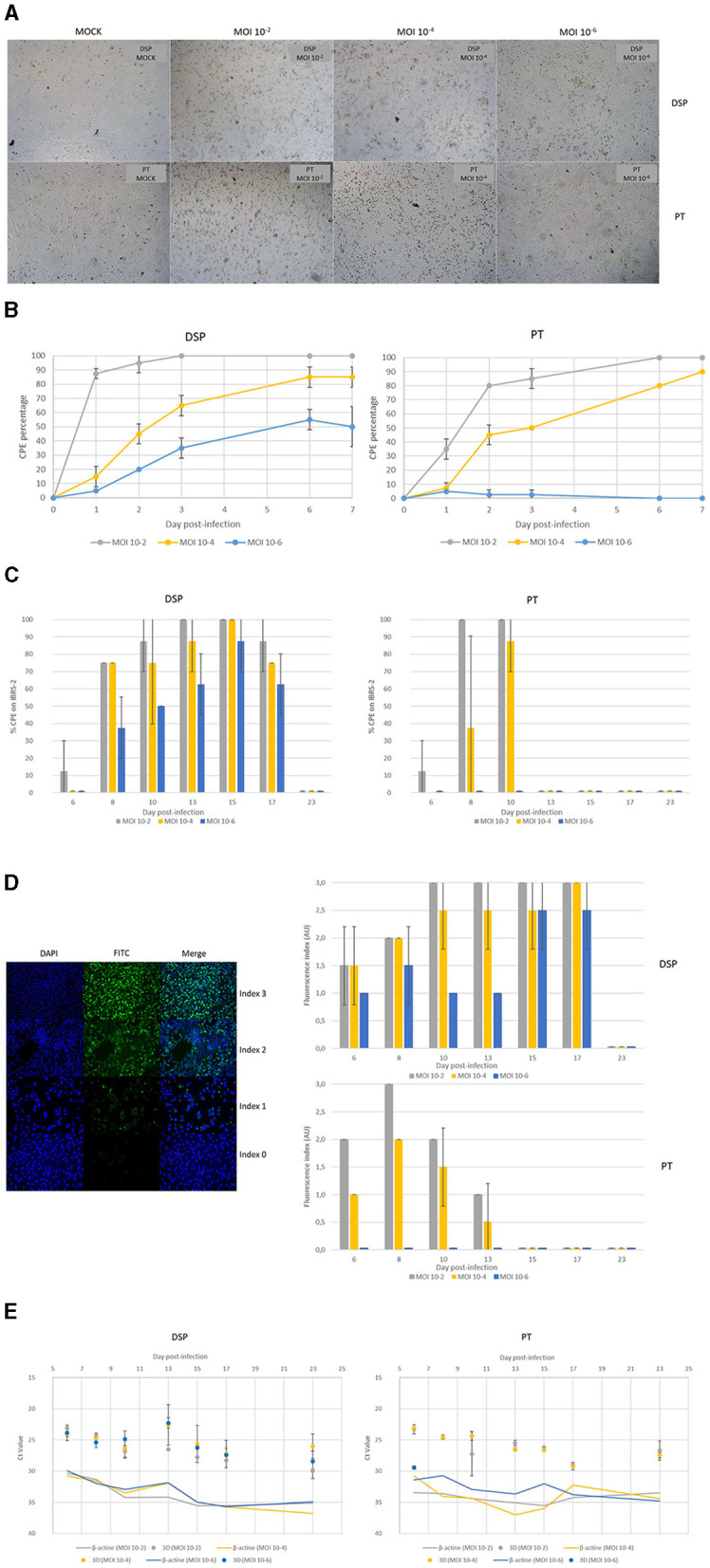
Monitoring of FMDV infection of ovine DSP and PT cultured in monolayers. Ovine DSP and PT were cultured in monolayers and were infected with FMDV O Cl2.2 at MOI 10^−2^, 10^−4^, 10^−6^, or a placebo conditioned medium. Viral inoculum has not been removed from the inserts until the first sample collection. **(A)** CPE observation at 48 hpi. **(B)** CPE evolution during the first seven dpi. CPE was visually estimated using brightfield microscopy. **(C)** Infectious FMDV detection by viral isolation. Viral isolation on IBRS-2 sensitive cells were performed using collected supernatants to detect infectious FMDV. CPE on IBRS-2 after a 48 h incubation with supernatants were visually estimated. The results presented here concern culture supernatants collected during the first 23 dpi. **(D)** FMDV 3D^pol^ antigen detection by immunofluorescence. IBSR-2 96-well plates used for viral isolation were fixed with 4% paraformaldehyde and then permeabilized with Triton X-100 before being stained with specific mouse 3D^pol^ antibody and a goat anti-mouse Alexa 488 (green). Cell nuclei were stained with Hoechst (blue) and cells were visualized by fluorescence microscopy using a DMi8 microscope. Results were arbitrarily classified according to an index ranging from 0 to 3. 0 indicates no fluorescence, 1 a small amount of fluorescent cells, 2 a majority of fluorescent cells and 3 indicates that almost all cells are fluorescent. Evolution of fluorescence levels estimated after 48h of incubation of IBRS-2 with culture supernatants harvested up to 23 dpi. Results presented here are the average of two samples. **(E)** FMDV 3D^pol^ RNA detection by rtRT-PCR. Duplex rtRT-PCR targeting 3D^pol^ FMDV RNA as well as β-actin housekeeping gene was performed. The results presented here concern results from culture supernatants collected during 23 dpi.

According to light microscopy observation, FMDV O/FRA/1/2001 Clone 2.2 infection of ovine DSP and PT monolayers resulted in a variable cell lysis depending on both the MOI used during infection and the infected cell population (Figures 2A, B). Cells from DSP appeared to be more sensitive to early infection as cytopathic effect (CPE) on these cells was almost complete after only 3 days post-infection at MOI 10^−2^, while it took 6 days for PT cells. Cytopathic effect also appeared faster in DSP infected at MOI 10^−4^ (65% CPE after 3 days) than in PT (50% CPE after 3 days). However, at this MOI CPE observed on DSP has reached a stagnation phase at 6 dpi, while CPE observed on PT was still increasing after 6 dpi. Furthermore, while MOI 10^−6^ appeared to be sufficient to effectively infect the DSP, this was not the case for the PT that showed only an extremely limited CPE that disappeared within the first 6 days post-infection. Despite some phases of cell growth recovery (data not shown), no complete reconstitution of the monolayer was observed after its destruction. Throughout the experiment, no CPE was detected for either the DSP or PT MOCK samples.

### 3.3 No infectious FMDV detected beyond 17 days post-infection on ovine DSP monolayers

Cell culture supernatants collected during the whole experiment were tested for the presence of infectious viruses by inoculation on FMDV-sensitive IBRS-2 cells. FMDV-induced CPE on IBRS-2 was estimated by microscopy observation (Figure 2C). The MOCK samples did not exhibit any CPE. The results again depend on both the population of infected cells and the MOI used for the infection. Indeed, CPE which indicated the presence of infectious FMDV particles, was detected in supernatants collected from 6 dpi to 17 dpi for DSP infected at the three different MOI, with an associated-CPE percentage correlated to the inoculum concentration. Regarding PT supernatant analysis, CPE was observed on IBRS-2 cells from day 6 to day 10 when PT were infected at MOI 10^−2^, and only from day 8 to day 10 for MOI 10^−4^. No infectious virus was detected in MOI 10^−4^ infected PT supernatants collected between 6 dpi and 23 dpi.

IBRS-2 cells used for viral isolation were then fixed and stained to detect FMDV 3D^pol^ antigens by IF (Figure 2D). There was no discernible fluorescence associated with MOCK samples. Once more, results are widely influenced by the MOI applied as well as the population of infected cells. 3D^pol^ antigens were detected in IBRS-2 incubated with DSP supernatants collected up to 17 dpi for MOI 10^−2^, 10^−4^, and 10^−6^. Similarly to what has been observed for the detection of infectious viruses, it appears that the proportion of 3D^pol^ antigen positive cells is correlated to the concentration of the inoculum used. Regarding IBRS-2 incubated with supernatants obtained from FMDV infection of PT cells, 3D^pol^ antigens were detected only for MOI 10^−2^ and 10^−4^, up to 13 dpi. Globally, the proportion of 3D^pol^ antigen positive cells is higher after incubation with supernatants from DSP infection than after incubation with supernatants from PT infection.

Throughout the experiment, cell culture supernatants from DSP and PT infection were analyzed for the presence of viral RNA by rtRT-PCR targeting the 3D^pol^ protein coding region of FMDV. The housekeeping-gene control β-actin was detected at Ct values between 29 and 37 for DSP and from 29 to 39 for PT. FMDV 3D^pol^ was not detected in the cell culture supernatant prior to infection, as well as in MOCK samples, but was detected at 6 days after infection at MOI 10^−2^, 10^−4^, and 10^−6^ for both cell populations (Figure 2E). Foot-and-mouth disease virus 3D^pol^ RNA was detected by rtRT-PCR in DSP supernatants collected from 6 dpi to 23 dpi for the DSP cells infected regardless of the MOI. 3D^pol^ RNA Ct values ranged between 23 and 30 Ct in supernatants of DSP infected at MOI 10^−2^, between 24 and 28 Ct in supernatants of DSP infected at MOI 10^−4^ and from 22 to 29 Ct for MOI 10^−6^. Regarding PT supernatants, 3D^pol^ RNA was detected from 6 to 23 dpi when infected at MOI 10^−2^ and 10^−4^, with Ct values ranging from 23 to 29 Ct. In contrast, when infected at MOI 10^−6^, 3D^pol^ RNA detection was only possible in supernatant from day 6 post-infection. No RNA was detected in the other samples.

### 3.4 Primary ovine DSP cells grew in multiple layers at the air-liquid interface

**Figure 3:**
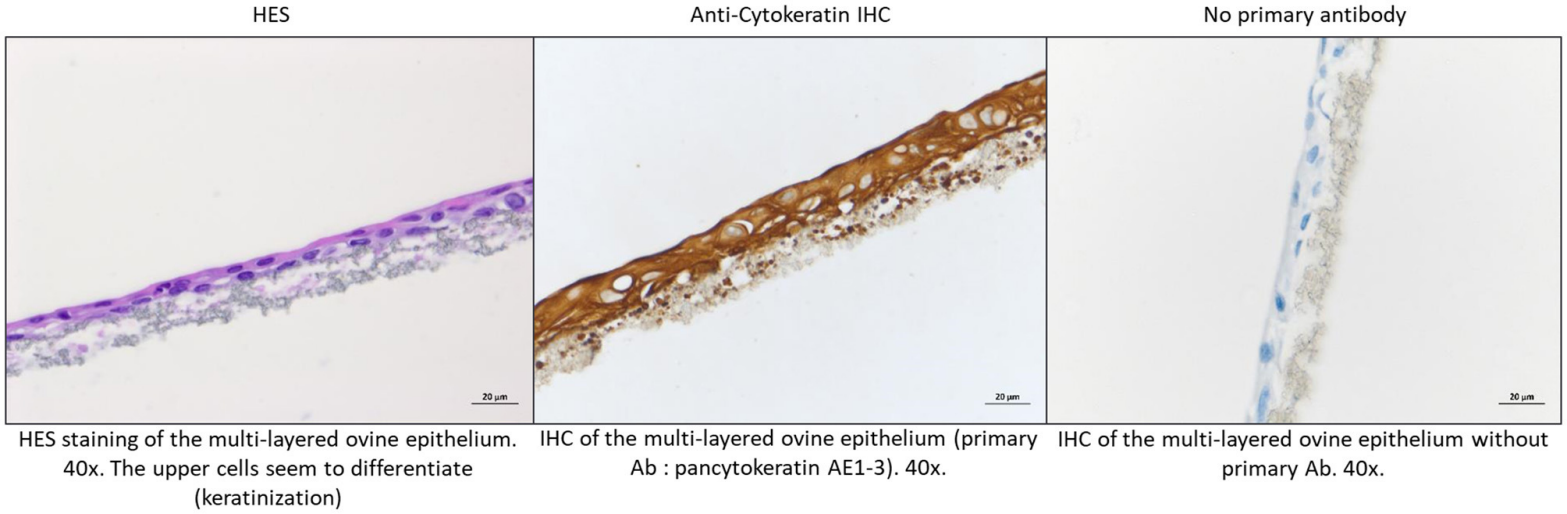
DSP cell multilayers characterization. DSP were cultured in multilayers at the air-liquid interface during 6 weeks. Some inserts were sacrificed to further investigation. HES staining. To this purpose, inserts were fixed in formalin and then stained with HES. Stained inserts were observed by microscopy to estimate the number of cell layers. Cytokeratin staining. IHC characterization was also performed to assess cytokeratin expression in DSP cells cultured at the air-liquid interphase.

In order to study the cooperation that may exist between the layers forming an epithelium, DSP and PT were grown in multilayers cultured at the ALI to mimic a natural epithelium. The DSP cells managed to develop correctly on the collagen-coated membrane, but it was not the case for the PT cells. After 5 weeks in culture at the ALI, the DSP were organized into thin bilayers dotted with clusters made of a more numerous layers of cells, as shown by HES staining of the inserts (Figure 3). Cytokeratin immunohistochemistry detection performed on inserts indicates that cells grown on the inserts still express this epithelial marker (Figure 3). Therefore, only the DSP cells multilayers were then infected. Infection was monitored as it was previously done for monolayers.

### 3.5 Infectious FMDV detected beyond 28 dpi on ovine DSP multilayers

**Figure 4:**
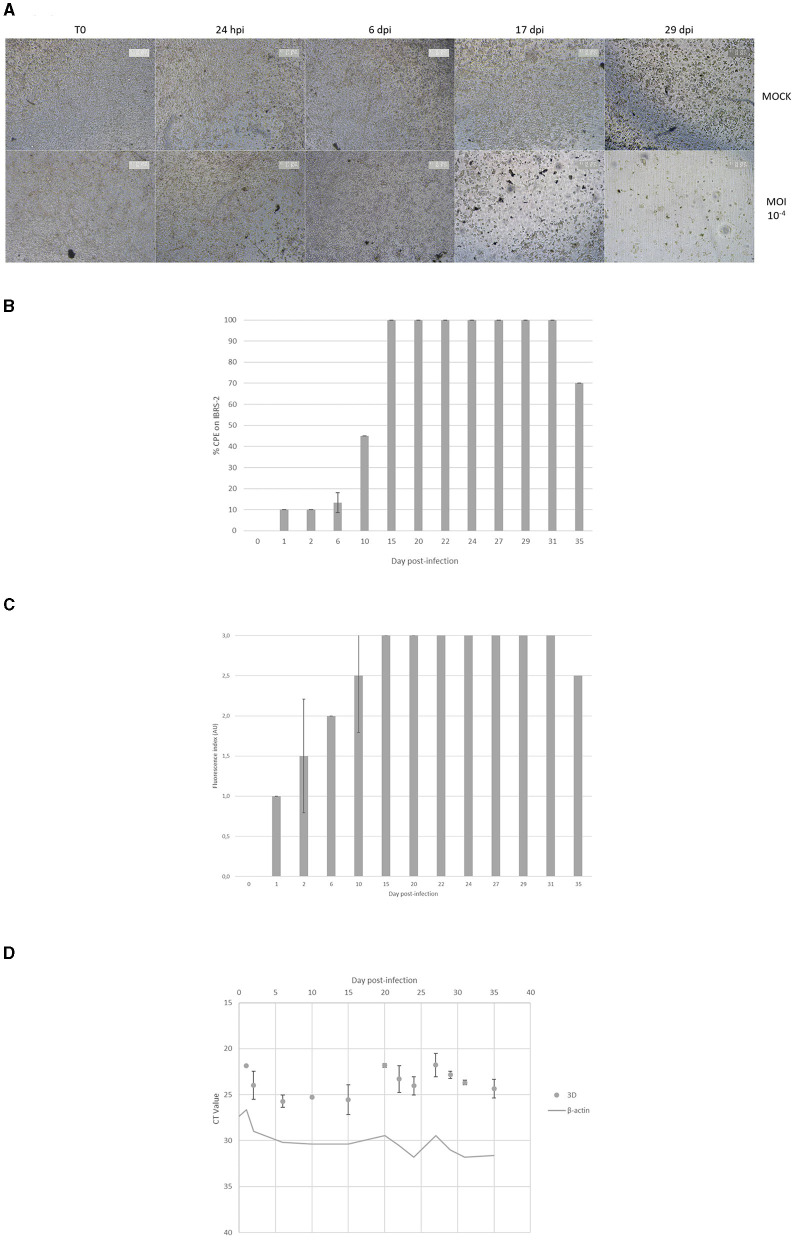
Monitoring of FMDV infection of ovine DSP cultured in multilayers at the air-liquid interface. DSP were cultured in multilayers at the air-liquid interface during 5 weeks before being infected with FMDV O Cl2.2 at MOI 10^−4^, or a placebo conditioned medium. CPE were visually estimated almost daily during the first 2 weeks post-infection. **(A)** CPE evolution on DSP multilayers. Microscopic CPE observation was pictured just before infection, at 24 hpi, 48 hpi, 7 dpi and 14 dpi for infected and MOCK condition. **(B)** Infectious FMDV detection by viral isolation. Viral isolation on IBRS-2 sensitive cells were performed using collected upper supernatants to detect infectious FMDV. CPE on IBRS-2 after a 48 h incubation with supernatants passaged once in IBRS-2 were visually estimated. The results presented here concern culture supernatants collected during 35 dpi. **(C)** FMDV 3D^pol^ antigen detection by immunofluorescence. IBRS-2 96-well plates used for viral isolation were fixed with 4% paraformaldehyde and then permeabilized with Triton X-100 before being stained with specific mouse 3D^pol^ antibody and a goat anti-mouse Alexa 488. Cell nuclei were stained with Hoechst and cells were visualized by fluorescence microscopy using a DMi8 microscope. Results were arbitrarily classified according to an index ranging from 0 to 3. 0 indicates no fluorescence, 1 a small amount of fluorescent cells, 2 a majority of fluorescent cells and 3 indicates that almost all cells are fluorescent. Here is presented the evolution of fluorescence levels estimated after 48 h of incubation of IBRS-2 with culture supernatants. The results presented here concern culture supernatants collected during 35 dpi. **(D)** FMDV 3D^pol^ RNA detection by rtRT-PCR. Duplex rtRT-PCR targeting 3D^pol^ FMDV RNA as well as β-actin housekeeping gene was performed. The results presented here concern culture supernatants collected during 35 dpi.

According to light microscopy observations, the FMDV O/FRA/1/2001 Clone 2.2 infection of DSP multilayers at MOI 10^−4^ resulted in a gradual CPE. Thus, relatively few differences could be observed between infected and MOCK samples during the first days after infection, and then the layers of infected inserts were progressively degraded until they were almost destroyed after 29 dpi. No evidence of cell recovery has been observed until the end of the infection monitoring (Figure 4A).

Throughout the entire process, cell culture upper supernatants were collected and examined for the presence of infectious viruses on IBRS-2 cells. After 48 hpi, no CPE was found on IBRS-2 incubated with the MOCK supernatants. As shown in Figure 4B, CPE was observed on IBRS-2 cultured in presence of upper supernatants collected from day 1 to day 35 post-infection. Average CPE percentage was estimated about 10% when IBSR-2 were incubated during 48 h with supernatants from day 2 post-infection and 45% for the ones from 10 dpi. A CPE close to 100% was visualized when IBRS-2 cells were cultured in presence of supernatants harvested from 15 dpi to 31 dpi. A 70% CPE was associated after viral isolation on supernatants collected at 35 dpi.

After the viral isolation, IBRS-2 cells were fixed and stained to detect 3D^pol^ antigens. There was no discernible fluorescence related to MOCK samples. IBRS-2 incubated with upper supernatants harvested during the first 2 days after infection showed a small proportion of 3D^pol^ antigen positive cells (between 10% and 50%), classified as index 1 or 2 (Figure 4C). Around half of the IBRS-2 cells incubated with supernatants obtained at 6 dpi were positive for the 3D^pol^ antigen (index 2). The supernatants collected from 15 dpi to 35 dpi were incubated for 48 h, and the wells under observation showed a massive majority of marked cells (index 2.5–3).

Insert upper supernatants were also examined for the presence of viral RNA using rtRT-PCR targeting the 3D^pol^ protein coding region of FMDV. The housekeeping gene control β-actin was found at Ct values ranging from 29 to 33. No FMDV RNA was detected in the insert upper supernatant harvested prior to infection or from MOCK samples. 3D^pol^ RNA was detected in infected inserts supernatants collected throughout the experiment, i.e. up to 35 dpi, as shown in Figure 4D. The measured Ct values ranged from 21 to 27 with no real decrease in the amount of 3D^pol^ RNA detected over time.

## 4 Discussion

The existing gaps of knowledge regarding FMDV persistence, can be partially explained by a lack of suitable cell models. While primary Bovine Thyroid cells (BTY) have long been considered as the best available tool for FMDV isolation, the associated difficulties of supply and culture have promoted the use of cell lines mostly derived from kidney epithelium, tissues of limited relevance with respect to the pathophysiology of FMD (22). Since bovine and porcine models particularly suited to FMDV studies was developed (18, 20), using primary cells from the dorsal soft palate, we considered it relevant to develop a similar model from the anatomically relevant sites in sheep, namely the PT and DSP.

Primary epithelial cells from ovine PT and DSP were therefore isolated and cultured. Cells from both sites of primary FMDV replication in this species were successfully cultured. These cells were then maintained in culture and tested in freeze-thaw resistance assays. The epithelial character of the cultured DSP and PT cells was confirmed through morphological observations and the immunofluorescence detection of selected epithelial markers. Thus, we have shown that DSP and PT look-like epithelial cells, and that they express cytokeratin, a constitutive protein of intermediate filaments (23, 24). The mild expression of cytokeratin in DSP cells is consistent with their vimentin expression, characteristic protein of fibroblastic cells but which can also be found in epithelial cells, and potentially related to FMDV survival (25). Vimentin synthesis has typically been linked to the epithelial-mesenchymal transition (EMT) (21, 26, 27). It is not surprising that vimentin was found more largely than cytokeratin because these markings were carried out after numerous passages; indeed, it is known that this transition affects primary epithelial cells over passages (28).

Anti-integrin αVβ6 staining of ovine DSP cells showed that these cells significantly express the preferential receptor for FMDV, indicating their probable susceptibility to infection by this virus (29). The susceptibility of DSP and PT cells to type-O FMDV infection was assessed on monolayers. Thus, we were able to successfully infect these two cell populations, which appear to be almost as susceptible as the bovine DSP cultured by Hägglund et al. (18). Hence, whereas the bovine DSP infected at MOI 10^−2^ showed an almost complete CPE at 24 hpi, the CPE observed at 24 hpi is close to 90% for the ovine DSP and is considered complete after only 3 dpi. However, this experiment also showed that PT were slightly less susceptible to early FMDV infection than DSP as it took more than 3 dpi to observe a CPE above 90%. Furthermore, it appeared that infection of PT at the lowest MOI (MOI 10^−6^) was very quickly interrupted and only induced an extremely limited CPE. Regardless of the MOI used to infect PT and DSP, we were able to detect infectious FMDV in the culture supernatants by viral isolation on susceptible IBRS-2 cells. Consistent with what was observed in terms of infectious virus, infection at MOI 10^−6^ led to the detection of viral antigens in the supernatants harvested up to 17 dpi for DSP but not in the supernatants of PT cells. Regarding viral RNA detection, we were able to demonstrate the presence of 3D^pol^ RNA up to 35 dpi in the culture supernatants from the DSP cells, with very weak Ct values variations between the three MOI. A similar observation was made regarding 3D^pol^ RNA detection in the culture supernatants of PT cells infected at MOI 10^−2^ and 10^−4^. In contrast, infection of these cells at 10^−6^ MOI revealed viral RNA only up to 5 dpi. Infection of DSP cells at MOI 10^−2^, 10^−4^, and 10^−6^ and PT cells at MOI 10^−2^ and 10^−4^ with FMDV thus resulted in the detection of infectious virus as well as viral antigens and viral RNA in the culture supernatants harvested above 28 dpi, the limit at which the persistence of FMDV is now defined. However, as the persistence of FMDV is a phenomenon involving a certain balance between the presence of the virus and the survival of its host, it is difficult to affirm that we developed an ovine cellular model allowing the study of persistent infection since the cell cultures were destroyed by the virus during the infection and no sign of reconstitution was observed.

According to the findings from monolayer cultures, DSP cells are slightly more susceptible to FMDV infection than PT cells, so we chose to focus our investigation exclusively on DSP cells. To establish a stratified epithelium, these cells were cultivated at the air-liquid interphase. Our multilayer cultures histological characterization supports the development of a stratified epithelium. Cytokeratin characterization by immunohistochemistry performed on DSP multilayers confirmed their epithelial nature. After infection with FMDV at MOI 10^−4^, multilayers of DSP cells exhibited a progressive CPE, which was less severe than that observed in monolayers, but still resulted in almost complete destruction of the cells at 29 dpi. Observed CPE seemed stronger than the limited CPE observed when the bovine model was infected at MOI 10^−2^ (18). While the monitoring of the bovine model infection allowed us to observe a complete reconstitution of the cell layer over time, this was not the case for the ovine model. Indeed, despite some phases of reconstruction of pieces of the cell layer, it was continuously degraded by the virus. Analysis of upper culture supernatants collected during DSP multilayer infection revealed the presence of infectious viruses, viral antigen and viral RNA up to 35 dpi. It thus appears that the presence of infectious virus is more durable in the multilayer model than in monolayers. Regarding the moderated CPE in the multilayers, it seems that the cooperation between the different cell layers could delay the infection of the lower layers and promote the establishment of a long-term infection (19). However, it is again difficult here to state that the model developed in this study allows the establishment of a persistent infection by FMDV since, as we observed during the infection of primary ovine cells in monolayers, the cell layer is almost fully destroyed. Since these attempts to establish a persistent infection were carried out using DSP cells from a single animal, it would seem appropriate to try again using tissues collected from other individuals. An attempt should also be made to establish such an infection in cells from the palatine tonsils, the structures most likely to be persistently infected.

Despite this, in this study we established two primary cell populations, from the palatine tonsils and dorsal soft palate of sheep, which are susceptible to acute and long-term infection with FMDV (4, 11). The establishment of an air-liquid interphase cultured multilayer model from ovine DSP allows to reproduce natural conditions by mimicking a stratified epithelium sensitive to FMDV infection. As a result, it can be used in combination with the bovine model established by Hägglund et al. (18) and a porcine model based on DSP cells (20) to further explore the FMDV differential persistence, with special emphasis on how epithelial innate immune responses are conserved from one species to another during infection. The use of these multilayered models will help to reduce the number of studies involving animals by enabling hypotheses to be evaluated in biological models that are more appropriate than monolayers of non-specific lineage cells.

Given the very limited number of currently available *in vitro* models to study FMDV infection in sheep, it might be interesting to immortalize these cells to produce the first ovine tissue-derived cell line of interest for the study of FMDV. Provided they are sensitive to the other serotypes of FMDV and able to induce a complete immune response, such a cell line has the potential to be used for either FMDV research or diagnosis.

## Data availability statement

The raw data supporting the conclusions of this article will be made available by the authors, without undue reservation.

## Ethics statement

Ethical approval was not required for the study involving animals in accordance with the local legislation and institutional requirements because sampling was performed on dead animals used for another experiment for which ethical approval was given.

## Author contributions

MS: Conceptualization, Investigation, Methodology, Writing – original draft. EL: Conceptualization, Investigation, Methodology, Writing – review & editing. ARe: Investigation, Writing – review & editing. ARo: Investigation, Writing – review & editing. CB-C: Investigation, Writing – review & editing. A-LS: Investigation, Writing – review & editing. HH: Investigation, Writing – review & editing. SH: Conceptualization, Investigation, Methodology, Writing – review & editing. J-FV: Conceptualization, Investigation, Methodology, Writing – review & editing. LB: Conceptualization, Supervision, Writing – review & editing. SB-B: Conceptualization, Investigation, Methodology, Supervision, Writing – review & editing.

## References

[1] Knight-Jones TJD Rushton J. “The economic impacts of foot and mouth disease – What are they. How big are they and where do they occur?” Prev Vet Med. (2013) 112:161–73. 10.1016/j.prevetmed.2013.07.013 23958457 PMC3989032

[2] Jamal SM Belsham GJ. Foot-and-mouth disease: past, present and future. Vet Res. (2013) 44:116. 10.1186/1297-9716-44-116 24308718 PMC4028749

[3] Kitching RP. Clinical variation in foot and mouth disease: cattle: -EN- -FR- -ES.-*Rev Sci Tech OIE*. (2002) 21:499–504. 10.20506/rst.21.3.1343 12523690

[4] Arzt J Baxt B Grubman MJ Jackson T Juleff N Rhyan J. The pathogenesis of foot-and-mouth disease II: viral pathways in swine, small ruminants, and wildlife; myotropism, chronic syndromes, and molecular virus–host interactions. Transbound Emerg Dis. (2011) 58:305–26. 10.1111/j.1865-1682.2011.01236.x 21672184

[5] Stenfeldt C Arzt J. The Carrier Conundrum; a review of recent advances and persistent gaps regarding the carrier state of foot-and-mouth disease virus. Pathogens. (2020) 9:167. 10.3390/pathogens9030167 32121072 PMC7157498

[6] World Organization for Animal Health (OIE). Chapter 3.1.8: Foot and Mouth Disease (Infection with Foot and Mouth Disease Virus). In: Manual of Diagnostic Tests and Vaccines for Terrestrial Animals. (2021). Available online at: https://www.oie.int/fileadmin/Home/eng/Health_standards/tahm/3.01.08_FMD.pdf (accessed April 11, 2022).

[7] Arzt J Belsham GJ Lohse L Bøtner A Stenfeldt C. Transmission of foot-and-mouth disease from persistently infected carrier cattle to naive cattle via transfer of oropharyngeal fluid. mSphere. (2018) 3:e00365–18. 10.1128/mSphere.00365-18 30209130 PMC6135961

[8] Fish I Stenfeldt C Spinard E Medina GN Azzinaro PA Bertram MR. Foot-and-mouth disease virus interserotypic recombination in superinfected carrier cattle. Pathogens. (2022) 11:644. 10.3390/pathogens11060644 35745498 PMC9231328

[9] Childs K Jackson B Harvey Y Seago J. Trans-encapsidation of foot-and-mouth disease virus genomes facilitates escape from neutralizing antibodies. Viruses. (2022) 14:1161. 10.3390/v14061161 35746633 PMC9229618

[10] Van Bekkum JG Frenkel HS Frederiks HHJ Frenkel S. Observations on the carrier state of cattle exposed to foot-and-mouth disease virus. Tijdschr Diergeneeskd. (1959) 84:1159–64.

[11] Burrows R. The persistence of foot-and mouth disease virus in sheep. J Hyg. (1968) 66:633–40. 10.1017/S0022172400028369 4303955 PMC2130666

[12] Stenfeldt C Pacheco JM Smoliga GR Bishop E Pauszek SJ Hartwig EJ. Detection of foot-and-mouth disease virus RNA and capsid protein in lymphoid tissues of convalescent pigs does not indicate existence of a carrier state. Transbound Emerg Dis. (2016) 63:152–64. 10.1111/tbed.12235 24943477

[13] Gray A Wood B Henry E Azhar M King D Mioulet V. Evaluation of cell lines for the isolation of foot-and-mouth disease virus and other viruses causing vesicular disease. Front Vet Sci. (2020) 7:426. 10.3389/fvets.2020.00426 32851014 PMC7401924

[14] Kopliku L Relmy A Romey A Gorna K Zientara S Bakkali-Kassimi L. Establishment of persistent foot-and-mouth disease virus (FMDV) infection in MDBK cells. Arch Virol. (2015) 160:2503–16. 10.1007/s00705-015-2526-8 26215440

[15] Torre JC Dávila M Sobrino F Ortín J Domingo E. Establishment of cell lines persistently infected with foot-and-mouth disease virus. Virology. (1985) 145:24–35. 10.1016/0042-6822(85)90198-9 2990100

[16] De Castro MP. Clonal variation in the swine kidney cell line, IB-RS-2, in relation to morphology karyotype and susceptibility to the foot-and-mouth disease virus (FMDV). Arqs Inst Biol, S Paulo. (1970) 37:103–27.

[17] Kasza L Shadduck JA Christofinis GJ. Establishment, viral susceptibility and biological characteristics of a swine kidney cell line SK-6. Res Vet Sci. (1972) 13:46–51. 10.1016/S0034-5288(18)34087-6 4336054

[18] Hägglund S Laloy E Näslund K Pfaff F Eschbaumer M Romey A. Model of persistent foot-and-mouth disease virus infection in multilayered cells derived from bovine dorsal soft palate. Transbound Emerg Dis. (2020) 67:133–48. 10.1111/tbed.13332 31419374 PMC7003861

[19] Pfaff F Hägglund S Zoli M Blaise-Boisseau S Laloy E Koethe S. Proteogenomics uncovers critical elements of host response in bovine soft palate epithelial cells following *in vitro* infection with foot-and-mouth disease virus. Viruses. (2019) 11:53. 10.3390/v11010053 30642035 PMC6356718

[20] Sarry M Bernelin-Cottet C Michaud C Relmy A Romey A Salomez A-L. Development of a primary cell model derived from porcine dorsal soft palate for foot-and-mouth disease virus research and diagnosis. Front Microbiol. (2023) 14:1215347. 10.3389/fmicb.2023.1215347 37840704 PMC10570842

[21] Yang J Antin P Berx G Blanpain C Brabletz T Bronner M. Guidelines and definitions for research on epithelial–mesenchymal transition. Nat Rev Mol Cell Biol. (2020) 21:341–52. 10.1038/s41580-020-0237-9 32300252 PMC7250738

[22] Brehm KE Ferris NP Lenk M Riebe R Haas B. Highly sensitive fetal goat tongue cell line for detection and isolation of foot-and-mouth disease virus. J Clin Microbiol. (2009) 47:3156–60. 10.1128/JCM.00510-09 19656987 PMC2756941

[23] Kuburich NA den Hollander P Pietz JT Mani SA. Vimentin and cytokeratin: good alone, bad together. Semin Cancer Biol. (2022) 86:816–26. 10.1016/j.semcancer.2021.12.006 34953942 PMC9213573

[24] Wang C-C Jamal L Janes KA. Normal morphogenesis of epithelial tissues and progression of epithelial tumors. Wiley Interdiscip Rev Syst Biol Med. (2012) 4:51–78. 10.1002/wsbm.159 21898857 PMC3242861

[25] Gladue DP O'Donnell V Baker-Branstetter R Holinka LG Pacheco JM Fernández Sainz I. Foot-and-mouth disease virus modulates cellular vimentin for virus survival. J Virol. (2013) 87:6794–803. 10.1128/JVI.00448-13 23576498 PMC3676138

[26] Guarino M. Epithelial–mesenchymal transition and tumour invasion. Int J Biochem Cell Biol. (2007) 39:2153–60. 10.1016/j.biocel.2007.07.011 17825600

[27] Kalluri R Weinberg RA. The basics of epithelial-mesenchymal transition. J Clin Invest. (2009) 119:1420–28. 10.1172/JCI39104 19487818 PMC2689101

[28] Pieper FR Van de Klundert FA Raats JM Henderik JB Schaart G Ramaekers FC. Regulation of vimentin expression in cultured epithelial cells. Eur J Biochem. (1992) 210:509–19. 10.1111/j.1432-1033.1992.tb17449.x 1459133

[29] Monaghan P Gold S Simpson J Zhang Z Weinreb PH Violette SM. The Alpha(v)Beta6 integrin receptor for foot-and-mouth disease virus is expressed constitutively on the epithelial cells targeted in cattle. J Gen Virol. (2005) 86(Pt 10):2769–80. 10.1099/vir.0.81172-0 16186231

